# Cumulative Effects of Multiple Modifiable Risk Factors on Cardiovascular Disease Mortality

**DOI:** 10.3390/jcm15041321

**Published:** 2026-02-07

**Authors:** Jonathan Yunzhou Xiong, Kai Goldenstein Vonkiel, Lance Ridpath, Chris Wood, Jill Cochran

**Affiliations:** Department of Clinical Sciences, West Virginia School of Osteopathic Medicine, 400 Lee Street, Lewisburg, WV 24901, USA; kgoldensteinvonkiel@osteo.wvsom.edu (K.G.V.);

**Keywords:** cardiovascular disease, death rate

## Abstract

**Background/Objectives**: The study evaluated whether the cumulative burden of multiple, modifiable risk factors worsened cardiovascular disease (CVD) mortality in West Virginia. **Methods**: This retrospective observational study looked at all CVD mortality rates per county of West Virginia between 2011 and 2020 and each county’s modifiable risks. Income, ratio of people per PCP (PCP Ratio), and Food Desert Status (FDS), and the death rate from the Census Bureau, AMA, US Department of Agriculture, and the West Virginia Department of Health and Human Services (WV HHS) were collected, respectively. Multiple linear regression and ANOVA analyses were performed for each combination of factors towards the death rate. **Results**: Multiple linear regression demonstrated that combined risk factors were more strongly associated with CVD mortality than any single factor alone. Over the 10-year period, West Virginia’s population aged overall. Each individual risk factor was significantly associated with CVD mortality (Income: F(1,537) = 53.39, *p* < 0.0001; PCP Ratio: F(1,537) = 21.49, *p* < 0.0001; FDS: F(1,537) = 5.13, *p* = 0.024). Two two-way interactions were significant (PCP Ratio:Income: F(1,537) = 22.03, *p* < 0.001; FDS:Income: F(1,537) = 5.23, *p* = 0.022), while the PCP Ratio:FDS interaction and the three-way interaction were not. **Conclusions**: Counties burdened by several modifiable risk factors experienced disproportionately elevated CVD mortality, exceeding what would be predicted by the additive effects of each risk factor individually. These findings highlighted the importance of addressing cumulative socioeconomic and healthcare access factors at the county level.

## 1. Introduction

Cardiovascular disease (CVD) remains the leading cause of death in the United States, affecting one in three adults as of 2020. CVD has a projected healthcare cost of $1.34 trillion by 2050 [1,2]. Between 2010 and 2022, over 10 million people died from CVD, and more alarmingly, from 2019 to 2022, the CVD death rate increased [3]. In 2022, 5399 West Virginians died from cardiovascular disease [4], and hospitals in West Virginia charged over a billion dollars for CVD hospitalizations [5]. There are many risk factors of cardiovascular disease; however, in recent years, there has been increasing interest in describing the distribution of modifiable risk factors [5,6].

Three factors have been frequently examined in relation to CVD death rates: income, primary care physician (PCP) ratio, and food desert status (FDS) [7,8,9]. These factors were picked because they are modifiable, and these could be changed at the population level. Low income has been consistently reported alongside higher cardiovascular disease death rates, with West Virginia being one of the poorest states [10,11,12,13]. For example, West Virginia’s poverty rate was 16.7%, which was significantly higher than the national poverty rate of 11.1% [14,15]. The state also has one of the highest proportions of its population who live in primary care health professional shortage areas (HPSA). Per the Kaiser Family Foundation’s 2024 Primary Care Health Professional Shortage Areas survey (KFF), approximately 748,000 people live in HPSAs [16].

Another significant factor that affects the CVD death rate is the availability of PCPs [8]. According to the American Medical Association (AMA), 83 million Americans reside in areas that do not have adequate access to PCPs [17]. Previous studies have reported lower CVD mortality rates in areas with higher PCP density [18]. In West Virginia, approximately 30% of the population have low access to preventive care services, and the state is expected to have a 14% physician shortage by 2030 [19].

Living within a food desert, an area of low-income that lacks supermarkets and grocery stores, has been associated with obesity, heart disease, and diabetes [20]. It is currently estimated that between 35.2 million and 83.5 million Americans live within a census tract with low access to grocery stores. In addition, adding a new supermarket may affect the overall health of the community. This may be because of the perceived access to healthy foods and the economic development it brings [21].

Although each of these variables has been independently associated with CVD death rates, examining them in isolation might have overlooked important interrelationships. Evaluating their interactions may provide deeper insight into how these factors relate, the strength of their associations, and where resources could be most effectively allocated.

The purpose of this retrospective observational study was to assess how county-level income, PCP Ratio, and food desert status, in combination, are associated with CVD mortality in West Virginia.

## 2. Methodology

Three factors were chosen: income, PCP ratio, and FDS. Between 2011 and 2020, all data points were collected exclusively from publicly accessible, publicly reported datasets. All information relating to the yearly CVD death rate was collected through public records by the West Virginia Department of Health and Human Services (WV HHS).

Income was obtained at the county level through the US Census Bureau’s yearly estimates report [22]; primary care physician access was measured as the number of people per PCP, i.e., PCP ratio. This was obtained through the AMA and Human Resources and Services Administration (HRSA) [18,23]. A county’s food desert status was calculated through the USDA’s Food Research Atlas [24]. As there is no standardized definition of a food desert at a county level, we assigned the label of “food desert” to a county if a simple majority of census tracts within a specific county were food deserts. As the USDA publishes their findings every 5 years (2015 and 2019), the research team decided that the data before and on the published year would be consistent with that year (i.e., 2011’s food desert status comes from the 2015 report). This definition of a food desert was utilized to cover scenarios where a county changed its status the year after the report’s release.

### Statistical Analysis

During the initial regression analysis, a Shapiro–Wilk test detected a lack of normality in the residuals. To correct this, a Box–Cox analysis showed that a transformation of x^−0.14^ on the income variable would alleviate normality issues. It should be noted that the x^−0.14^ transformation causes the reversal of the positive/negative signs in front of any regression coefficients involving income. There were no significant outliers in data as assessed by the visual inspection of Q-Q plots. An ANOVA test was conducted to confirm that income, PCP ratio, and FDS independently showed statistical significance towards CVD death rates. A multiple linear regression model with interactions was used to compare the predisposing factors to CVD death rate. Due to inconsistent access to patient medical records, age of death was unable to be determined.

Statistical significance was set at *p* < 0.05 (2-tailed). An effect is considered statistically significant at the α = 0.05 level if the value 0.0 is outside the interval. All analysis was performed in GraphPad Prism (10.4.1, GraphPad Software, Boston, MA, USA) and confirmed with SAS software (v9.4, SAS Institute Inc., Cary, NC, USA). All heatmaps were created in Quantum Geographical Information System (QGIS) (3.36.2-Maidenhead, QGIS Development Team (2024) and utilizing a projected, EPSG:26917-NAD83 (UTM zone 17N) Coordinated Reference System.

## 3. Results

A Shapiro–Wilk test showed no significant deviations from normality over the capture period (2011–2025 W = 0.9322, *p* = 0.5694; 2016–2020 W = 0.9395, *p* = 0.6345). The test was also run on the residuals of each risk factor, and it did not show a significant deviation from normality (W = 0.9956, *p* = 0.1239). In Supplemental Figures S1–S3, the Box–Cox transformed data was not included for real-world visualization. Non-transformed data is available in Supplementary Table S1.

The age and sex demographics for West Virginia are shown in Table 1. Because age is a known unmodifiable risk factor for CVD, an aging population is important context. Over the 10-year period, the overall population stayed similar in size (2011–2015: 1,851,907; 2016–2020: 1,805,945). While the overall population stayed stable, there was a significant increase in the proportion of individuals >65-year-old compared to the <65-year-old population over time (Odds Ratio = 1.1900, 95% CI [1.1837, 1.1963]).

Table 2 reflects the results of the multiple linear regression of the median income, PCP Ratio, and FDS. This study found that over the course of the capture period, there was a significant connection between each of the factors analyzed to the CVD death rate (Income: F(1,537) = 53.39, *p* < 0.0001; PCP ratio: F(1,537) = 21.49, *p* < 0.0001; FDS: F(1,537) = 5.126, *p* = 0.024). When analyzing the linear regression combinations, lower income combined with either a greater PCP Ratio or FDS two-way combinations were associated with higher CVD deaths (PCP Ratio + Income: F(1,537) = 22.03, *p* < 0.001; FDS + Income: F(1,537) = 5.226, *p* = 0.022). With respect to the two-way interaction, PCP Ratio:FDS, and the three-way interaction, no significant increase or decrease in CVD mortality was observed (*p* = 0.9242 and *p* = 0.9327 respectively).

Higher PCP ratio was positively associated with the outcome ((β = 0.42, 95% CI [0.2434, 0.6002]), indicating a mild increase in death rates. Being in a food desert (β = 1507, 95% CI [211, 2803]) and being poorer (β = 8227, 95% CI [6023, 10,431]) were both associated with worse prognosis. While the x^−0.14^ transformation necessitated a sign reversal for interpretation, the implications were clear: lower income significantly scales with increased CVD mortality. Additionally, there was a statistically significant interaction between PCP Ratio and Income (β = −1.88, 95% CI [−2.658, −1.092]). This indicates that the effect of PCP ratio on CVD is exacerbated in lower income counties. Similarly, a significant interaction between food desert status and income (β = −6667, 95% CI [−12301, −1033]) shows that the effect of being in a food desert on CVD is also exacerbated in lower income counties. No significant interaction was observed between PCP Ratio and food desert counties, nor for the three-way interaction, indicating no observable cumulative effect.

## 4. Discussion

While there have been multiple epidemiological studies showing correlations between each one of the investigated risk factors and CVD death rates, this is the only study that investigated how risk factors relating to economics, medical access, and geography may exert cumulative associations on CVD mortality. As CVD is a multifactorial disease, looking at multiple categories of risk factors helps contextualize the unique landscape of West Virginia. Supplemental Figures S1–S3 depict the average income, number of people per PCP, and CVD death rate per capita in each county (see Supplemental Section for county level breakdown of all variables). While there have been studies that showed correlations between income, PCP ratio, and food deserts on CVD mortality independently, there has not been any known study examining the interactions between these factors as it relates to CVD death rates [8,10,11,12,19,26]. Because West Virginia is one of the poorest states in the U.S., the factors chosen for this study were selected based on their expected sensitivity to socioeconomic influences [18,27].

The main findings of this study were that West Virginia’s median household income, PCP Ratio, and living in a food desert all significantly increased CVD deaths. When analyzing the interactions among the risk factors, counties that had a lower household income demonstrated stronger associations between lack of access to PCPs and food desert status to CVD mortality. These two-way interactions showed larger effects than those observed from each factor independently (Income:PCP Ratio Estimate = −1.875, 95% CI (−2.658, −1.092); Income:FDS Estimate = −6667, 95% CI (−12301, −1033)). These findings suggest that certain combinations of socioeconomic and healthcare access factors are associated with a disproportionate increase in CVD death rates, highlighting the cumulative effects that are not captured when modifiable risk factors are analyzed independently. Stacking multiple socioeconomic and health risk factors together has already shown an effect. Though the two-way interaction of PCP Ratio:FDS and the three-way interaction did not show significance, the small sample size of food desert counties (n = 2) may not have a high enough power to detect a meaningful difference. Because of this study’s county-level definition of food desert, only a small number of counties were classified as food deserts. As a result, all interactions with FDS were constrained to a limited subset of counties. This may have reduced statistical power and may have limited its ability to detect associations, increasing the risk of a false-negative finding.

Table 1 shows an increase in the overall age of West Virginia in 2020 compared to 2011. A pressing concern for many stakeholders, especially for the state, older adults have declining health and are poorer [28,29]. As age and sex are known unmodifiable risk factors to developing CVD, identifying trends in these factors are important [25,30]. Between the two time periods (2011–2015 and 2016–2020), the age distribution of the population in West Virginia shifted in a manner consistent with broader demographic aging trends. The significant demographic shift towards the >65-year-old cohort suggests an increased ‘at-risk’ pool that increases PCP utilization and increase strain on the medical infrastructure [31,32].

Although CVD mortality has declined in both West Virginia and the United States of America, the reduction in West Virginia was 0.8% smaller than the national average (WV: −6.2%; US: −7.0%) [33]. While national trends indicate a decline in US mortality due to better risk factor management, our results suggest that localized socioeconomic clusters in West Virginia are more insulated from these improvements, possibly due to the interaction of poverty and food access [34].

A major challenge in healthcare delivery in West Virginia is the predominance of rural counties. Notably, 13 counties each have a total population of fewer than 10,000 residents. These rural communities also tend to be the poorest. The higher mortality observed in rural, low-income counties suggests a spatial compounding of disadvantages, where geographic isolation and economic scarcity function as structural barriers to cardiovascular health.

The data suggests that better economic conditions are associated with lower CVD deaths. With a stronger economy, higher disposable income may facilitate the attraction of businesses and healthcare professionals. This increase may be associated with greater availability of grocery stores that offer healthy foods. The Lancet Regional Health—Americas observed that across the world, high-income countries on different continents have similar death rates, whereas lower-income countries had higher rates [35].

## 5. Limitations

One of the limitations that this study encountered was the limited, annually updated data from the federal government. For food deserts, county designations were retained until the next report was released because these reports are issued every 5 years. Without more granular data, yearly changes could have been missed. With a primary outcome of death due to a chronic condition, different factors may take varying amounts of time to manifest. Although factors such as income level may exert relatively immediate effects, this study may lack the ability to detect long-term effects such as access to preventive medicine. Future research should examine the temporal lag and the influence of age between changes in these factors and their impact on CVD mortality.

There are certain counties of West Virginia that suggest there is no apparent shortage of primary care physicians. However, many of these counties have very small populations, with some towns consisting of only a few hundred residents, which obscures the true limitations in access to care. Notably, there are three (3) medical schools within West Virginia: West Virginia University (Monongalia), West Virginia School of Osteopathic Medicine (Greenbrier), and Marshall University (Cabell and Wayne), all of which employ PCPs at higher rates compared to other areas. The higher density of PCPs around the medical schools could have also covered issues with healthcare access across the rest of the county.

The use of food deserts in this study and its interaction with other factors may be less generalizable because only a couple of counties were designated as a food desert. Because “food desert” is defined by the US Department of Agriculture at the census tract level but all other variables that were collected WV HHS at the county level, a change in categorization of a food desert was required to achieve a level of continuity. This was due to limited and variable data of income and the number of PCPs at the census tract level. Future studies conducted at the census tract level may yield higher sensitivity in detecting how food deserts influence CVD death rates.

There was a change in federal designations beginning in 2013 as to who is considered a PCP. Before, only those with MDs were counted. Afterwards, DOs were included and OBGYN specialists were removed [36,37]. This may cause an unrepresentative result of the PCP Ratio. We decided to keep 2011–2013 in this study as the standard for grants/funding and federal designations were consistent. Therefore, we do not anticipate that this definitional change substantially impacts the results of our analysis.

## 6. Conclusions

Although it is well known that socioeconomic and healthcare access factors affect CVD death rates, very few studies have looked at how multiple modifiable risk factors cumulatively affect CVD mortality. This study provides observations that county-level income, PCP availability, and food desert status are each independently associated with cardiovascular disease mortality in West Virginia, while also demonstrating that the certain combinations of these modifiable risk factors produce effects that exceed those expected from their additive contributions alone. These findings suggest that analyses focusing on single risk factors may underestimate cardiovascular risk in economically disadvantaged regions where multiple structural barriers coexist. Given that CVD is still the leading cause of death in the United States, this study methodology can be used as a blueprint to investigate other risk factors or use the same methodology for other chronic diseases with multifactorial risks.

## Figures and Tables

**Table 1:** Average age breakdown, sexual characteristic, race, and rurality of West Virginia divided into two (2) equal time groups from WV HHS [25].

		Average 2011–2015	Average 2016–2020
		n (%)	n (%)
Age (years)			
	<5	102,923.2 (5.56%)	95,787.4 (5.30%)
	5–17	279,128 (15.07%)	269,168.6 (14.90%)
	18–44	620,769.2 (33.52%)	589,398.8 (32.64%)
	45–64	529,284.4 (28.58%)	492,475.6 (27.27%)
	>65	319,572.0 (17.26%)	359,114.8 (19.89%)
Sex			
	Male	914,207.4 (49.37%)	894,189.8 (49.51%)
	Female	937,699.6 (50.63%)	911,755.2 (50.49%)
Race			
	White/Caucasian	1,743,908 (93.86%)	1,685,246 (93.32%)
	Black or African American	66,500 (3.58%)	66,601 (3.69%)
	Asian, Native Hawaiian, and other Pacific Islander	14,972.6 (0.81%)	15,423.2 (0.85%)
	American Indian and Alaska Native	4278.6 (0.23%)	4132.2 (0.23%)
	All Other Races	28,289 (1.52%)	34,539.2 (1.91%)
Rurality			
	Urban	902,810 (48.72%)	800,857 (44.64%)
	Rural	950,184 (51.27%)	992,859 (55.35%)

**Table 2:** Results from the multiple linear regression model between each factor and all combinations of factors as it relates to CVD death rate.

Variable	β	SE	95% CI	|t|
Intercept *	−1484	254.3	[−1984, −984.8]	5.84
PCP Ratio *	0.4218	0.0908	[0.2434, 0.6002]	4.64
FDS [Yes] *	1507	659.7	[211.0, 2803]	2.28
Transformed(Income) *	8227	1122	[6023, 10,431]	7.33
PCP Ratio:Transformed(Income) *	−1.875	0.3987	[−2.658, −1.092]	4.70
FDS [Yes]:Transformed(Income) *	−6667	2868	[−12301, −1033]	2.33
PCP Ratio:FDS [Yes]	<−0.001	0.0063	[−0.013, 0.0118]	0.095
PCP Ratio:FDS:Transformed(Income)	−0.0023	0.0276	[−0.05656, 0.05189]	0.085

Cardiovascular disease mortality. β = coefficient. SE = Standard Error. 95% CI = 95% Confidence Interval. *p* < 0.05 is considered significant and indicated by (*).

## Data Availability

All data collected for this article can be accessed through governmental agencies. For Income: https://www.census.gov/quickfacts/fact/table/US/SEX255223 (accessed on 8 March 2025); For the number of PCPs: https://mchb.tvisdata.hrsa.gov/Narratives/Overview/7f0c79bc-c4b9-49c7-9790-e21513c6db01#:~:text=Not%20surprisingly%2C%20WV%20consistently%20ranks,PCP)%20or%20health%20care%20provider (accessed on 8 March 2025) and https://doi.org/10.1001/jamainternmed.2018.7624; For Food Desert Status: https://www.ers.usda.gov/data-products/food-access-research-atlas/documentation (accessed on 6 April 2025); For cardiovascular disease death rates by year: https://dhhr.wv.gov/HSC/SS/Vital_Statistics/Pages/Vital_Statistics.aspx (accessed on 10 January 2025).

## Supplementary Materials

The following supporting information can be downloaded at: https://www.mdpi.com/article/10.3390/jcm15041321/s1, Table S1: Descriptive statistics of all the counties of West Virginia from 2011 to 2020 of all three (3) modifiable risk factors in the study; Figure S1: Median Household Income; Figure S2. Number of People per PCP; Figure S3. Average number of deaths per 100,000 due to cardiovascular disease.
