# Supplementary material for: Cumulative Effects of Multiple Modifiable Risk Factors on Cardiovascular Disease Mortality
Source: J Clin Med. 2026 Feb 7;15(4):1321. doi: 10.3390/jcm15041321 (PMC12941920; doi:10.3390/jcm15041321)
Supplement: Supplementary file 1 [file jcm-15-01321-s001.zip › jcm-4112078-supplementary.pdf]

Journal of
Clinical Medicine

MDPI

Supplementary Materials:

Table S1. Descriptive statistics of all the counties of West Virginia from 2011 to 2020 of all three (3) modifiable risk factors in the study. IQR = Interquartile Range.

		Median Income				
County Name	Mean (SD)		PCP		Death Rate (per 100k)	
Barbour	37118 (1787)	IQR	Mean (SD)	IQR	Mean (SD)	IQR
Berkeley	57473 (4328)	36285-38571	3008 (568.9)	2383-3350	334.6 (44.67)	288.2-378
Boone	40949 (2411)	53469-61090	2275 (119.7)	2205-2358	230.2 (35.39)	214.1-238
Braxton	36675 (5152)	38470-42760	3086 (543.5)	2731-3528	342.3 (42.25)	314.1-380.7
Brooke	46106 (3458)	31808-41653	2713 (951.2)	2228-2900	329.6 (48.19)	290.3-379.4
Cabell	38276 (1558)	42447-49069	4637 (725.5)	3995-4968	458.2 (47.06)	438.4-499.4
Calhoun	34397 (4571)	37597-38788	598.9 (71.45)	577.5-657.5	320.1 (27.17)	303-336.7
Clay	33208 (2067)	30583-38454	2923 (1642)	1990-3476	686.5 (968.7)	308.7-461.4
Doddridge	40556 (6526)	31541-35057	6946 (1994)	4856-8695	329.2 (59.48)	269.9-387.6
Fayette	37545 (3577)	34129-45771	6053 (1995)	4173-8234	273.5 (63.93)	230.5-329
Gilmer	37623 (3057)	34611-40633	1998 (295.5)	1843-2185	405.4 (19.09)	394.7-421
Grant	40771 (1418)	35600-39491	2433 (267)	2170-2673	504.3 (608.9)	277.6-383.9
Greenbrier	38917 (1804)	39778-41754	1715 (140.2)	1648-1763	414.3 (50.37)	371.4-468.5
Hampshire	34540 (8354)	37556-40214	826.6 (265.1)	683.5-765	386.8 (56.62)	344.3-401.5
Hancock	41709 (3748)	27938-42039	4013 (931.4)	3314-4663	313.8 (51.73)	276.9-353
Hardy	40029 (5808)	38484-45626	1574 (187.3)	1430-1718	460.9 (46.24)	409.3-497.7
Harrison	46140 (4479)	33903-46681	5671 (1510)	4337-6890	344.4 (50.98)	296-395.5
Jackson	43112 (3131)	42797-50858	891.4 (70.35)	862.5-966.8	386.4 (35.69)	350.5-416.1
Jefferson	70955 (6733)	40895-45547	3807 (2372)	2742-3225	372.8 (23.45)	353.7-390.6
Kanawha	46103 (843.7)	65299-77485	1934 (343.1)	1676-2060	238.5 (23.76)	223.7-259.2
Lewis	38270 (2700)	45588-46694	761.9 (27.74)	740-770	351.1 (24.58)	331.9-376.1
Lincoln	36825 (3256)	35944-39822	1708 (429.9)	1437-2088	445 (58.59)	374.7-493.7

Logan	37000 (646.3)	35132-38775	3480 (1188)	2300-4310	338.8 (52.67)	279.2-370.4
Marion	45545 (4356)	36484-37449	1907 (384.5)	1548-2210	417.4 (40.89)	385.8-442.9
Marshall	43303 (3511)	41821-49030	1721 (211.2)	1567-1930	405.9 (43.63)	363.1-449.3
Mason	40205 (4940)	40583-45931	2493 (357.2)	2270-2755	398.9 (29.81)	379.7-420.4
McDowell	24682 (1913)	37109-41780	1944 (283.3)	1767-2215	383.1 (34.46)	356.1-408.8
Mercer	37083 (2548)	22792-26191	2807 (907.5)	2093-3675	451.5 (63.47)	403.1-511.9
Mineral	38624 (8044)	34761-39708	1633 (146.9)	1520-1717	403.9 (33.85)	378.4-427.8
Mingo	33417 (1634)	31633-47250	3207 (376.1)	2948-3378	366.3 (37.01)	343-405.3
Monongalia	47172 (4374)	32157-34752	4138 (1695)	2860-5770	416.7 (44.29)	363.1-455
Monroe	38786 (2689)	43461-50558	736 (134.8)	702.5-818	203.8 (15.12)	188.4-213.4
Morgan	43393 (7713)	36636-40216	3014 (1666)	2245-2683	346.4 (73.72)	294.6-378.5
Nicholas	39422 (659.1)	36539-50932	2124 (296.1)	1940-2202	323 (57.9)	281.9-353
Ohio	43860 (3936)	38774-40070	1149 (74.68)	1108-1176	371.6 (23.09)	353.1-389.5
Pendleton	38073 (4243)	40631-48147	624 (58.34)	577.5-660	422.3 (57.5)	364.8-465.9
Pleasants	46258 (5792)	34153-41486	2374 (687.3)	1912-2575	411.7 (86.06)	333.2-465.2
Pocahontas	36393 (2900)	42190-50299	4485 (2378)	2531-7473	350.9 (69.36)	321.3-390.4
Preston	46935 (2912)	33807-38157	2975 (2378)	1836-2735	343.5 (63.79)	282.8-413
Putnam	57672 (3034)	45182-49210	3051 (726.1)	2528-3380	334.5 (32.33)	311.9-363.8
Raleigh	41411 (1620)	55668-59744	986.1 (109)	927.5-999.3	295 (36.73)	256.5-336.1
Randolph	39981 (2865)	40400-42610	1156 (81.24)	1087-1214	398.8 (52.13)	340-448.9
Ritchie	39709 (3809)	37332-41651	1299 (151)	1145-1463	394.7 (27.98)	372.5-420.7
Roane	32802 (4372)	35735-43765	4633 (1316)	3427-5026	375 (65.11)	337.3-412.8
Summers	35358 (1941)	28328-37513	2313 (664.6)	1822-2479	364.7 (49.39)	318.6-397.7
Taylor	44233 (4541)	33605-36931	2778 (627.3)	2306-3153	458.8 (74.2)	387-542.3
Tucker	41890 (4617)	39834-47548	2837 (558)	2440-3258	392.9 (33.33)	355.7-427.2
Tyler	40389 (3120)	37352-46123	2114 (321.2)	1741-2313	451.8 (103.5)	388.1-527.8

https://doi.org/10.3390/jcm15041321

Upshur	40100 (1252)	38485-41603	2449 (650)	2099-2884	386.6 (79.39)	346.1-417.6
Wayne	37917 (2231)	39333-40905	2042 (346.7)	1736-2300	317.2 (45.79)	300.2-337.9
Webster	30323 (3453)	36511-38460	1813 (211.3)	1625-1989	318.5 (33.01)	294.1-341.1
Wetzel	40061 (2450)	27231-33621	2772 (1153)	1799-4053	404.8 (54.85)	356.5-459.8
Wirt	39384 (3659)	37951-42371	2074 (356)	1805-2390	420.9 (56.18)	375.8-475.5
Wood	44310 (2423)	36988-41471	4914 (1359)	2913-5835	314.6 (40.86)	287.4-344.3
Wyoming	37504 (3531)	42280-46299	1232 (80.6)	1168-1289	401.5 (32.72)	376.6-423.9

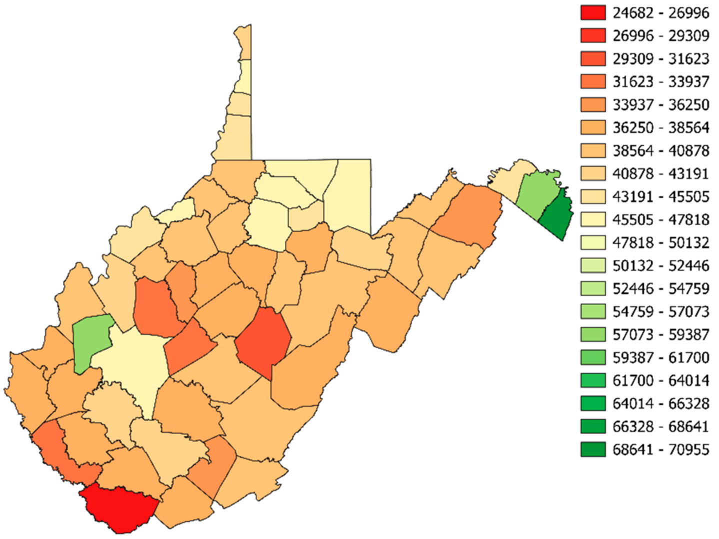

Figure S1. *Median Household Income. Purple outlines of counties represent counties that were considered food deserts at any point during the capture period.*

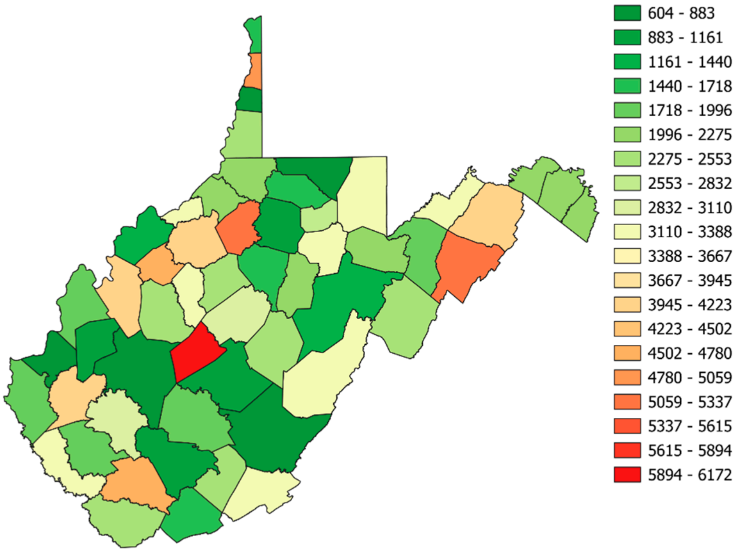

Figure S2. *Number of People per PCP. Purple outlines of counties represent counties that were considered food deserts at any point during the capture period.*

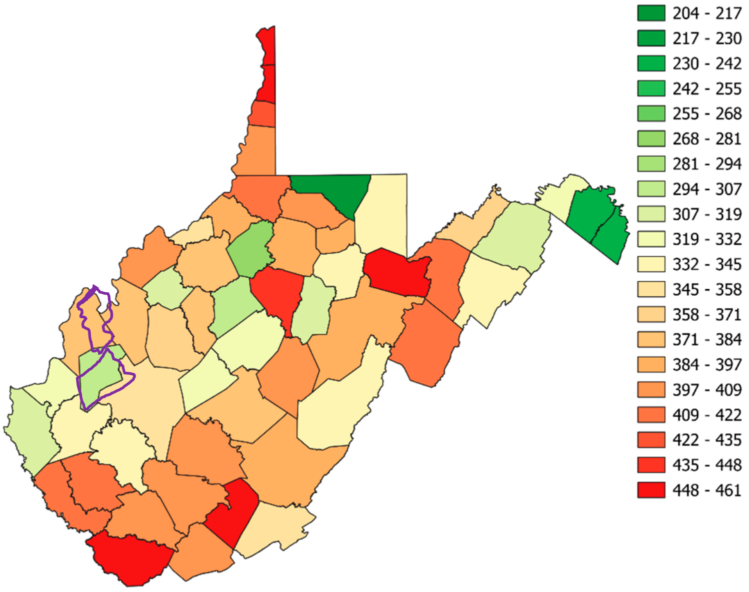

Figure S3. *Average number of deaths per 100,000 due to cardiovascular disease. Purple outlines of counties represent counties that were considered food deserts at any point during the capture period. Purple outlined counties indicate food desert status.:*

https://doi.org/10.3390/jcm15041321
